# Analysis of the impact of social determinants and primary care morbidity on population health outcomes by combining big data: A research protocol

**DOI:** 10.3389/fmed.2022.1012437

**Published:** 2022-12-16

**Authors:** Sabela Couso-Viana, Carmen Bentué-Martínez, María Victoria Delgado-Martín, Elena Cabeza-Irigoyen, Montserrat León-Latre, Ana Concheiro-Guisán, María Xosé Rodríguez-Álvarez, Miguel Román-Rodríguez, Javier Roca-Pardiñas, María Zúñiga-Antón, Ana García-Flaquer, Pau Pericàs-Pulido, Raquel Sánchez-Recio, Beatriz González-Álvarez, Sara Rodríguez-Pastoriza, Irene Gómez-Gómez, Emma Motrico, José Luís Jiménez-Murillo, Isabel Rabanaque, Ana Clavería

**Affiliations:** ^1^I-Saúde Group, South Galicia Health Research Institute (Instituto de Investigación Sanitaria Galicia Sur), SERGAS-UVIGO, Vigo, Spain; ^2^Department of Geography, Aragon University Environmental Sciences Research Institute (Instituto Universitario de Investigación en Ciencias Ambientales de Aragón/IUCA), University of Zaragoza, Zaragoza, Spain; ^3^Moaña Health Center, Vigo Area, SERGAS, Vigo, Spain; ^4^Health Promotion Service, Ministry of Health and Consumer Affairs, Public Health Research Group (Grup d’Investigació en Salud Pública/GISPIB), Balearic Islands Health Research Institute (IdISBa), Balearic Islands, Spain; ^5^La Jota Health Center, Aragonese Health Service, Aragon, Spain; ^6^Department of Pediatrics, Álvaro Cunqueiro Hospital, SERGAS, Vigo, Spain; ^7^Rare Diseases and Pediatric Medicine Group, South Galicia Health Research Institute (Instituto de Investigación Sanitaria Galicia Sur), SERGAS-UVIGO, Vigo, Spain; ^8^Department of Statistics and Operations Research, Biomedical Research Center (Centro de Investigacións Biomédicas/CINBIO), University of Vigo, Vigo, Spain; ^9^Galician Research and Mathematical Technology Center (Centro de Investigación e Tecnoloxía Matemática de Galicia/CITMAga), Vigo, Spain; ^10^Primary Care Management of Mallorca, Balearic Islands Health Research Institute, Balearic Islands, Spain; ^11^Department of Statistics and Operations Research, University of Vigo, Vigo, Spain; ^12^Network for Research on Chronicity, Primary Care and Health Promotion (Red de Investigación en Cronicidad, Atención Primaria y Promoción de la Salud/RICAPPS), Galicia, Spain; ^13^Balearic Islands Health Research Platform (Plataforma de Investigación en Información en Salud de Las Islas Baleares/PRISIB), Balearic Islands, Spain; ^14^Aragon Health Services Research Group (Grupo de Investigación en Servicios Sanitarios de Aragón/GRISSA), Aragon, Spain; ^15^Aragonese Institute of Health, Aragon, Spain; ^16^Department of Psychology, Loyola University Andalusia, Seville, Spain; ^17^Technical Sub-directorate for Information Management, Andalusian Health Service, Seville, Spain

**Keywords:** social determinants of health (MeSH), socioeconomic factors (MeSH), big data, electronic health records—HER, morbidity

## Abstract

**Background:**

In recent years, different tools have been developed to facilitate analysis of social determinants of health (SDH) and apply this to health policy. The possibility of generating predictive models of health outcomes which combine a wide range of socioeconomic indicators with health problems is an approach that is receiving increasing attention. Our objectives are twofold: (1) to predict population health outcomes measured as hospital morbidity, taking primary care (PC) morbidity adjusted for SDH as predictors; and (2) to analyze the geographic variability of the impact of SDH-adjusted PC morbidity on hospital morbidity, by combining data sourced from electronic health records and selected operations of the National Statistics Institute (*Instituto Nacional de Estadística/INE*).

**Methods:**

The following will be conducted: a qualitative study to select socio-health indicators using RAND methodology in accordance with SDH frameworks, based on indicators published by the *INE* in selected operations; and a quantitative study combining two large databases drawn from different Spain’s Autonomous Regions (ARs) to enable hospital morbidity to be ascertained, i.e., PC electronic health records and the minimum basic data set (MBDS) for hospital discharges. These will be linked to socioeconomic indicators, previously selected by geographic unit. The outcome variable will be hospital morbidity, and the independent variables will be age, sex, PC morbidity, geographic unit, and socioeconomic indicators.

**Analysis:**

To achieve the first objective, predictive models will be used, with a test-and-training technique, fitting multiple logistic regression models. In the analysis of geographic variability, penalized mixed models will be used, with geographic units considered as random effects and independent predictors as fixed effects.

**Discussion:**

This study seeks to show the relationship between SDH and population health, and the geographic differences determined by such determinants. The main limitations are posed by the collection of data for healthcare as opposed to research purposes, and the time lag between collection and publication of data, sampling errors and missing data in registries and surveys. The main strength lies in the project’s multidisciplinary nature (family medicine, pediatrics, public health, nursing, psychology, engineering, geography).

## Introduction

### Social determinants and health

Social determinants of health (SDH) are defined by the World Health Organization as, “*the conditions in which people are born, grow, work, live, and age, and the wider set of forces and systems shaping the conditions of daily life*” (1). Indeed, references to the influence of social and environmental factors on health and disease processes are already to be found in the Hippocratic Corpus, but these relationships did not assume special importance until the appearance of explanatory models in the latter part of the twentieth century, notably the model of health determinants used by the former Canadian Minister of Health, Marc Lalonde, in 1974 (2). In Spain, the reference model is the conceptual framework of the Determinants of Social Inequalities in Health drawn up and issued in 2010 by the Committee to Reduce Health Inequalities (3).

There is now solid evidence to show the influence of SDH on people’s health and wellbeing (4–6). Even so, it is important to ensure that special consideration be given to children, since it is in this period when many capacities are developed and go to form the basis of wellbeing over the course of a lifetime (7). Protecting children from adverse economic conditions reduces morbidity at adult age (8, 9).

The relationship between the COVID-19 pandemic and socioeconomic status has been reported in many countries. In Great Britain, persons living in underprivileged areas were observed to experience COVID-19 mortality rates that were twice as high as those of persons living in less deprived areas (10). During the first two waves of the pandemic in Barcelona, inequalities by age group, gender, geographic area, and income were clearly identified (11). Restrictive measures such as confinement, social distancing, restrictions on access to health centers, while possibly reducing the spread of infection, specifically impact persons who experience financial difficulties, limiting their activity and ability to ensure healthy nutrition, potentially increasing tobacco or alcohol use, or even hindering care in situations of domestic violence and favoring overcrowding in the home.

In recent years, different tools have been developed to facilitate analysis of SDH and apply this to health policy. In 2010, the WHO published the Urban Health Equity Assessment and Response Tool (Urban HEART), an instrument designed to assess and respond to health inequalities in urban areas (12). In 2017, the tool was implemented and adapted in Barcelona, tailored to the national context and shown to be of practical use (13). This guideline is based on indicators of key health outcomes and the main social determinants grouped into four policy domains (physical and infrastructural environment, human and social development, economy, and governance) (12).

A number of initiatives have proposed indicators which quantify social, wellbeing, and sustainability aspects related with health and the progress of societies (14). In this respect, one of the most important landmarks was the Stiglitz-Sen-Fitoussi (SSF) Report (15), which not only set the lines of subsequent research, but also laid the foundations for the main European surveys and statistics on the topic. In Spain, the National Statistics Institute (*Instituto Nacional de Estadística/INE*) applies a methodology similar to that used by EUROSTAT, in surveys such as the census (16) and the Living Conditions Survey (17). An experimental statistical exercise that draws directly on this latter report and seeks to analyze income with a level of breakdown by census section, is the “Atlas of Distribution of Household Income,” included in the 2021 Census (18).

In parallel, the health administrations of various countries are creating sets of basic indicators for the purpose of having multidimensional executive information, containing social determinants. This is the case of both Canada (*Indicateurs comparables de la santé*) and the United Kingdom (Key Statistics NHS). In the case of Spain, mention should be made of the National Health System (NHS) key indicators (19), the MEDEA project (20), and more recently, the Deprivation Index (21).

### Milestones in the analysis of morbidity with large databases

The MesH term “electronic health record (EHR)” was introduced in 2010, with an exponential growth in PubMed entries (currently standing at 26,236), as a consequence of the obvious advance in information and communication technologies. Some organizations are notable for having demonstrated the impact and challenges of its application to the study of health services and health policies.

In 2007, within the context of its Effective Health Care Program, the Agency for Health Care Research and Quality published its first guideline, “Registries for Evaluating Patient Outcomes: A User’s Guide.” The 4th edition, issued in 2020, along with a wide-ranging series of reports (22), have since become reference manuals, providing guidance on best practices for the design, functioning, analysis, and evaluation of patient records. A registry that is properly designed, built, implemented and analyzed, will provide unique scientific information on the effectiveness, safety and quality of any given medical service or intervention being studied. While the use of existing data sources has many advantages, the challenges of interoperability persist, and the use of these data introduces new considerations regarding their planning, accessibility, integration, etc. In conjunction with these technological changes, registries have been adapted to respond to the surge in research into patient-centered outcomes and the growing recognition that patients should be at the center of clinical research studies.

Since 1989, the Manitoba Center for Health Policy and Evaluation has had -and continues to have- intense academic activity and constant interaction with the health authorities and the community. The brunt of its research has focused on health determinants and health service delivery from a population perspective. E. Shapiro, N.P. Roos, L. Lix, among many authors, have published seminal papers on the application of administrative data to research and decision-making from a population stance (23).

Recent years have also witnessed a considerable growth, especially in Europe, in the use of registries as a method of generating new scientific evidence in primary care (PC). Practice-based research networks (PBRN) in Holland, USA, United Kingdom and Israel are an interesting instrument for changing the research culture and clinical practice of PC health professionals (24). PBRN are networks of general practitioners and other health professionals who seek to collaborate on projects focusing on practical problems and issues, thereby making for a constant exchange between practice and research. In Europe, these networks are grouped into the European General Practice Research Network, which was created in 1971 under the auspices of WONCA Europe, and has facilitated the systematic development of research on family medicine and PC across Europe (25).

The leading source for analyzing PC morbidity in Spain is the Primary Care Clinical Database (*Base de Datos Clínicos de Atención Primaria/BDCAP*), a large database that systematically collects anonymized and normalized clinical data from a sample of 4.7 million users assigned to NHS PC teams. The 2018 NHS Annual Report (26) shows that every person attended at PC health centers presents with a mean of 7.8 health problems: the breakdown by sex shows a mean of 6.9 active problems in the case of men and 8.6 in the case of women. Moreover, the registered number of health problems per person in PC changes with income level, employment status, and country of birth. A social gradient is in evidence, whereby the lower the income level, the higher the number of health problems, across all age groups and in both sexes. Unemployed persons register more health problems than do economically active persons (27).

Health services in Spain’s Autonomous Regions (ARs) have progressively embarked on an analysis of EHRs in ongoing projects at different stages of development. Among such projects, mention should be made of the Information System for Research in Primary Care (*Sistema de información para la investigación en Atención Primaria/SIDIAP*), conceived with the aim of exploiting EHRs in Catalonia, which contain a certain amount of anonymized clinical information on each citizen ascribed to a health center (28), and the *BIGAN* big data platform operated by the Aragon Health Service.

In the hospital sphere, the Specialized Care Activity Register (*Registro de Actividad de Atención Especializada*/*RAE-CMBD*), which pools information pertaining to the Minimum Basic Data Set (MBDS) for discharges from acute care hospitals, is the principal database for ascertaining morbidity in these types of hospitals (27). There are many studies in Spain that analyze the Specialized Care Activity Register, since it is linked to funding based on clinical complexity (Diagnosis-Related Groups) and requires the use of standardized, structured coding in hospitals (29, 30). There are many other examples, both by medical specialty and overall (31), including the Ministry of Health patient safety indicators (19).

The possibility of generating predictive health outcome models that combine a wide array of socioeconomic indicators with health problems is an approach to which growing attention is being paid. In this connection, the Personalized Medicine Platform was recently launched by the Carlos III Institute of Health, bringing together the Consortium Centers for Biomedical Research (*Consorcio Centro de Investigación Biomédica en Red/CIBER*) and health services countrywide: it seeks to identify and follow up a cohort of 200,000 individuals, along with their recorded data, adopting a holistic view of persons.

In view of the shift in scientific thinking outlined above and the advances made in health service technology and communications infrastructures, there are opportunities for linking real-world data and surveys, with the ensuing possibility of performing an innovative low-cost analysis by combining multiple socioeconomic indicators with PC morbidity. At the same time, the impact on health outcomes, including hospital morbidity and mortality, could likewise be quantified.

There has been ample coverage of the role of PC in prophylaxis of infections, prevention or delay of cardiovascular events and preventable conditions, and early detection and treatment of diseases that can benefit from this, with the resulting saving, not only financial, but also in terms of suffering, harm, hospital admissions, and quality of life. Similarly noteworthy are analyses of preventable hospitalizations (32) and studies on the use of hospital emergencies (33). This, coupled with aspects such as feasibility and comparability, led us to choose hospital morbidity as the outcome variable in this study.

We propose to analyze how social determinants influence PC morbidity, and this, in turn, influences population health outcomes such as hospital morbidity, by combining data from EHRs and *INE* statistical operations. In addition to predictive models, analysis by geographic area would doubtless be an extremely helpful element when it comes to planning activities and social and health resources. We also seek to analyze the impact adjusted for large groups of diseases (somatic and mental diseases, accidents and poisoning, COVID-19, chronic diseases), adjusted for the adult and pediatric populations, and adjusted for emergency and non-emergency admissions.

Health-prevention and health-promotion activities and/or distribution of resources may thus be a joint reflection of the needs and idiosyncrasies of individuals and their environment (34).

### Qualitative study to select socio-health indicators

Cross-sectional observational study with RAND methodology (35), a two-round modified Delphi technique, which will be carried out electronically. The panel of experts will consist of 15–20 persons reflecting research experience, academic experience, gender balance, and a broad professional spectrum.

In preparation for the meeting of experts, the study protocol and informed consent document will be circulated. By way of a framework, the determinants of social inequalities in health will be furnished (3, 36), taking into account the structural determinants (socioeconomic and political context, and axes of inequality) and intermediate determinants of health inequalities (material resources, psychosocial factors, behavioral and biologic factors, and health services) for prioritization of the indicators (37). Indicators will be presented for each of the domains previously identified from among the following *INE* surveys; Atlas of Health Determinants in Spain (38); Municipal Voters Roll (39); Urban Audit Indicators (40); Population and Housing Census (16); Deprivation Index (41); Atlas of Household Income Distribution (18): Atlas of Urban Vulnerability (42); Residential Building Atlas (43); and Statistical Atlas of Urban Areas (44).

The group members will evaluate the indicators twice. In the first round, the experts will score each of the indicators, by rating their degree of agreement on a Likert scale scored from 1 (strongly disagree) through 9 (strongly agree), along with the indicator’s suitability for measuring the dimension in question. A comments section will be included so as to allow the experts to add suggestions or observations. In the second round, the members’ own results, together with the aggregated results of the group, and their comments in a free text field, will be circulated. The indicators will then be scored again, and those whose median is above 7, without disagreement, will be selected. The process will be managed using the eDelphi software program (45).

### Quantitative study combining large databases

Health service EHRs, made up of all clinical data sets containing information relevant for healthcare purposes, will be used on an individualized basis. Any person who has been attended at least once in the NHS has an electronic record containing a note of any action (s) taken. For study purposes, it will be necessary to combine two large databases, drawn from different settings, to enable hospital morbidity to be ascertained, i.e., PC EHRs and the MBDS for hospital discharges.

This information will be aggregated and linked with various socioeconomic indicators, selected in the previous stage and sourced from microdata published by the *INE*. Every individual with an active PC episode will thus be linked to indicators selected from his/her own geographic unit.

Study period: In the case of PC morbidity, active episodes from 01/01/2016 through 31/12/2019 will be included. In the case of hospital morbidity, the following will be considered: general hospital morbidity, discharges from 01/01/2017 through 31/12/2019; hospital morbidity due to COVID 19, discharges from 01/01/2020 through 31/12/2020. The availability of socioeconomic indicators tends to vary, depending upon their publication by the *INE*.

#### Study scope and population

According to the 2018 Voters Roll, the participating ARs (Aragon and the Balearic Isles) have 2,491,478 inhabitants.

#### Inclusion/Exclusion criteria

The following will be included: for analysis of morbidity, all patients, pediatric and adult, having an active PC EHR at baseline; for analysis of determinants, indicators furnished as microdata by the *INE* and selected by the panel of experts.

#### Sample size

In the participating ARs, clinical data have been registered in the PC EHRs of their respective health services for a minimum of ten years. A total of 93.31% of the population has a NHS digital clinical history and is assigned to a given health center in accordance with the NHS service portfolio.

#### Variables and measuring instruments

Outcome variable: Hospital morbidity (somatic and mental diseases, accidents and poisoning, COVID-19, chronic diseases). We will consider the principal diagnosis at discharge, as shown in the MBDS with ICD-9-CM or ICD-10 coding, by Autonomous Region. Somatic diseases will be categorized differently by age group. In the adult population (over 14 years of age): Infections, Neoplasms, Digestive, Blood, Immune System, Cardiovascular, Locomotor, Nervous, Respiratory, Skin and Skin Appendages, Endocrine, Urinary, Genital/Breast. In the pediatric population (ages 0 through 14 years): Infections, Congenital Anomalies, Neoplasms, Digestive, Blood, Immune System, Eyes, Locomotor, Nervous, Respiratory, Skin and Skin Appendages, Endocrine, Urinary, Genital/Breast. In the case of chronic diseases, O’Halloran’s classification will be applied (46).

Independent variables: sex (women/men) in 5-year groups, age, nationality, copayment, geographic unit (Autonomous Region, province, town, basic health area, census section), PC morbidity, and indicators selected in the previous stage related with the dimensions identified by the Determinants of Social Inequalities in Health. All active episodes in PC EHRs will be selected. The International Classification of Primary Care (ICPC-2) will be used, excluding the R codes (symptoms, signs and abnormal clinical and laboratory findings, not elsewhere classified). The ICPC-2 coding was designed by selecting diseases and disorders having a prevalence of over 5% in PC, and has a much higher degree of aggregation than does the ICD-10. To be able to exploit morbidity as a whole, it is necessary to use a single classification on which the remaining classifications converge. Given that the ICD-9-CM (like the ICD-10) is, on the whole, a far more comprehensive and detailed classification than the ICPC-2, it follows that the base classification for a joint exploitation must necessarily be the latter. Accordingly, this calls for a unidirectional conversion from the fullest (ICD-9-CM or ICD-10) to the most condensed classification (ICPC). These equivalents have been published by the Ministry of Health.

#### Statistical analysis

Predictive models will be used for the impact on hospital morbidity. In this type of model, the initial sample will be divided into a random training sample (70%), with which the models used will be fitted, and the rest of the data (30%), which will be used as a test sample for validation of such models. In particular, multiple logistic regression models with penalizations (least absolute shrinkage and selection operator/LASSO) will be used, which will allow us to choose the most important variables in the studies undertaken. This study seeks to obtain an estimator of the probability of hospital admission or death, based on “relevant” information yielded by all the abovedescribed predictors. At this point, it will be essential to use the penalizations mentioned above, in order to prevent possible overfitting and obtain “simple” models based on the really important variables. The outcome variable is of the categorical type (yes/no). For greater ease of interpretation of results, well-known measures, such as the odds ratio and its corresponding confidence interval, will be used for detection of significant effects. To test the goodness-of-fit of models, we will use Nagelkerke’s R2, which measures the proportion of the variance in health outcomes explained by the selected predictors. In addition, aspects of the model’s performance, including calibration and discrimination, will also be studied. Calibration will be assessed using Brier’s score and plotting the non-parametric estimate of the association between observed outcome frequencies and predicted probabilities. To validate the model’s predictive capacity, the Receiver Operating Characteristic (ROC) curve and corresponding area under the ROC curve (AUC) will be used. To correct for possible optimism in the AUC values obtained, a training sample will be used to fit the models, along with another test sample, independent of the former, in which the AUC-test will be calculated on the basis of the relevant predictions.

To estimate the extent of inequalities across social class, two indices of socioeconomic inequality in health will be computed, i.e., the Relative Index of Inequality (RII) and the Slope Index of Inequality (SII) (47–49). A log-binomial regression model will be applied with the log link function for calculating the RII and the identity link function for the SII between the health outcomes and social variables identified in the consensus stage.

For analysis of geographic variability, penalized mixed models will be used, taking geographic unit as random effects (with subanalyses for each of the possible classifications, such as census section, town, AR). The following will be considered as fixed effects: Age, sex, PC morbidity, and socioeconomic indicators. The response variable of interest, hospital morbidity (with Poisson distribution), will be included in the model. Variables will be selected using the Backward Stepwise Regression method based on the Akaike Information Criterion (AIC). The parsimony of the different models obtained will be compared with the anova function, and will be validated by examining the pertinent diagnostic plots obtained with the residuals, to ascertain whether there are deviations from the hypotheses assumed by these types of models, such as normality, homoscedasticity, and absence of atypical values.

All the statistical analyses will be performed with the R statistical software package using the BayesX, rms, lme4 and epiDisplay packages. These packages are available free of charge from http://cran.r-project.org. The free software qgis^1^ will be used for spatial representation.

## Discussion of the study

The main limitations of collecting data on the basis of EHR pertain to the fact that the data have been collected for healthcare as opposed to research purposes (50). In line with the paper by Verheij et al. (50), we can contend that, in Spain, the use of such data in research is well founded. The use of EHRs is not only widespread, but the public health system covers almost the entire population (51). Although there is some variability between the records in the various ARs, the differences are not substantial, in that their systematization is regulated by law and that much of the information is unified by means of the NHS Health Information System (52). Despite this inter-regional variability, it should be noted that, within each AR, the processes are highly systematized through the use of software that unifies the records of all health professionals in the system. Furthermore, the extensive use in Spain of standardized classifications for many records (e.g., ICD-10 or ICPC), clinical practice guidelines, and protocols that seek to unify and update the clinical practice of all professionals on the basis of scientific evidence, greatly enhances comparability (50). It should also be stressed here that EHR systems have been implemented in Spain for over 10 years (52) and that training and refresher courses are held for health professionals.

The main limitations of the *INE*’s statistical operations are: the time lag between collection and publication of data; sampling or non-response errors (both controlled and analyzed); interviewer bias (in the case of the census, this is controlled for by having group coordinators who supervise the work); and underrepresentation in surveys of people who prove difficult to locate at a permanent place of abode. Despite these limitations, the data made publicly available by the *INE* offer great advantages, such as their high degree of comparability at both a national and European level, and their homogeneity across time, since the concepts and basic methodology remain unaltered over long periods. Age and geographic unit at baseline will be considered, something that will introduce a bias due to measurement error.

By way of strengths, special mention should be made of the integration of data sourced from two health services with several *INE* surveys. This aspect renders the multidisciplinary nature of the project obligatory, i.e., clinicians, epidemiologists, experts in operations research, geography, and information and communication technologies. Furthermore, it will enable comparison of different machine learning models, such as regression models, random forest or deep learning (53), and geographic regressions.

The panel of experts will be made up by 15-20 experts. In their review about consensus methods, (54), Murphy et al. state that when combining individual judgments, more is generally better. As the number of judges increases the reliability of a composite judgment increases. In a theoretical study which assumed errors of judgment around a “true” value, it was found that under most sets of assumptions, there was little advantage in terms of “group validity” in increasing numbers much above ten. Recently (55), the average number of experts included was usually in the low to medium double-digit range (e.g., ID1: median = 17 invited experts; ID11: mean = 40 experts in the first Delphi round). However, it is not the number of participants but the whole reporting of the method what matters most (56).

Insofar as the RAND methodology is concerned, the appropriateness criteria and quality indicators designed with its application would seem to possess both construct and predictive validity (57). Moreover, it is recognized by leading institutes, such as the NICE (National Institute for Health and Care Excellence) in the United Kingdom or the HAS (*Haute Autorité de Santé*) in France, as an appropriate consensus method for comparison of complex processes.

Lastly, the extraction of data from the various web platforms will be performed by technical staff specifically engaged to manage such platforms in each AR, and will be brought into line with a data-management plan. To analyze the information, a specific platform will be developed, with a single server and shared desktop for researchers, and access to the database in line with standardized procedures.

The results of this evaluation are relevant, not only for professionals who manage social, educational or health service data systems, but also for scientists who explore high dimensional social data.

## Data availability statement

The original contributions presented in this study are included in the article/supplementary material, further inquiries can be directed to the corresponding author.

## Author contributions

SC-V, CB-M, EC-I, MD-M, and AC contributed to the conception and design of the study and participated in the drafting of the manuscript. ML-L, AC-G, MR-Á, MZ-A, AG-F, PP-P, RS-R, BG-Á, IG-G, EM, JJ-M, and IR contributed to the conception and design of the study, and participated in the critical review of the manuscript. AC-G and MD-M consisted the pediatrics team. AC, SC-V, and MD-M were in charge of project implementation and follow-up: this included, inter alia, the engagement of professionals, translation of project documentation, submission to the ethics committee, and identification of training needs. MZ-A and AC maintained international collaboration. JR-P, MR-Á, SR-P, and AC were responsible for the statistical analysis. All agreed to assume responsibility for all aspects of the study. All authors read and approved the final manuscript.

## Footnotes

1
https://www.qgis.org/
